# Supplementary material for: 1‐Undecene from Pseudomonas aeruginosa is an olfactory signal for flight‐or‐fight response in Caenorhabditis elegans
Source: EMBO J. 2021 Jun 4;40(13):e106938. doi: 10.15252/embj.2020106938 (PMC8246062; doi:10.15252/embj.2020106938)
Supplement: Supplementary file 1 — Appendix [file EMBJ-40-e106938-s002.docx]

**1-Undecene from *P. aeruginosa* is an olfactory signal for flight or fight response in *C. elegans***

Deep Prakash^1^, Akhil MS^1^, Buddidhathi Radhika^2^, Radhika Venkatesan^2,3^, Sreekanth H. Chalasani^4^, Varsha Singh^1,^ *

^1^Department of Molecular Reproduction Development and Genetics, Indian Institute of Science, Bangalore INDIA

^2^National Center of Biological Sciences, Bangalore, INDIA

^3^Department of Biological Sciences, Indian Institute of Science Education and Research, Mohanpur, INDIA

^4^Salk Institute for Biological Studies, La Jolla, USA

* Lead Contact: [varsha@iisc.ac.in](mailto:varsha@iisc.ac.in)

**This file contains:**

**Appendix Figures S1-S4 and legends** (Pages 2-8)

**Appendix Table 1** (pages 9-10)

**Appendix Figure S1 (related to Figure 2)**

**
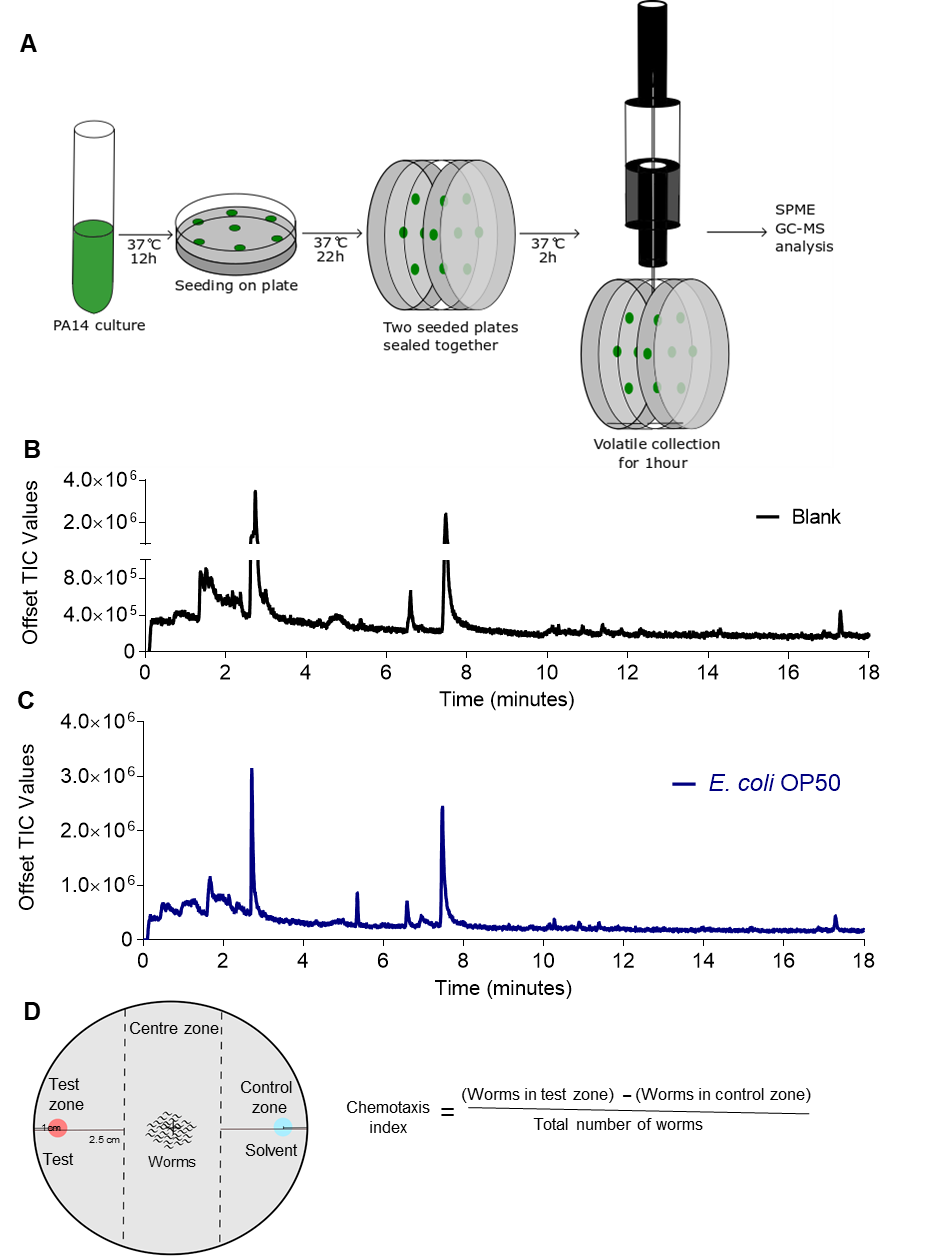
Appendix Figure S1. (Related to figure 2).**

(A) Schematic for preparation of bacterial headspace sample for SPME GC-MS/MS analysis.

(B-C) GC-MS/MS profile of volatiles produced by (B) Blank, media control (C) 24 h old lawn of *E. coli* OP50.

(D) Schematic of chemotaxis assay for attractant and repellent chemical.

**Appendix Figure S2 (related to Figure 2 and 3)**


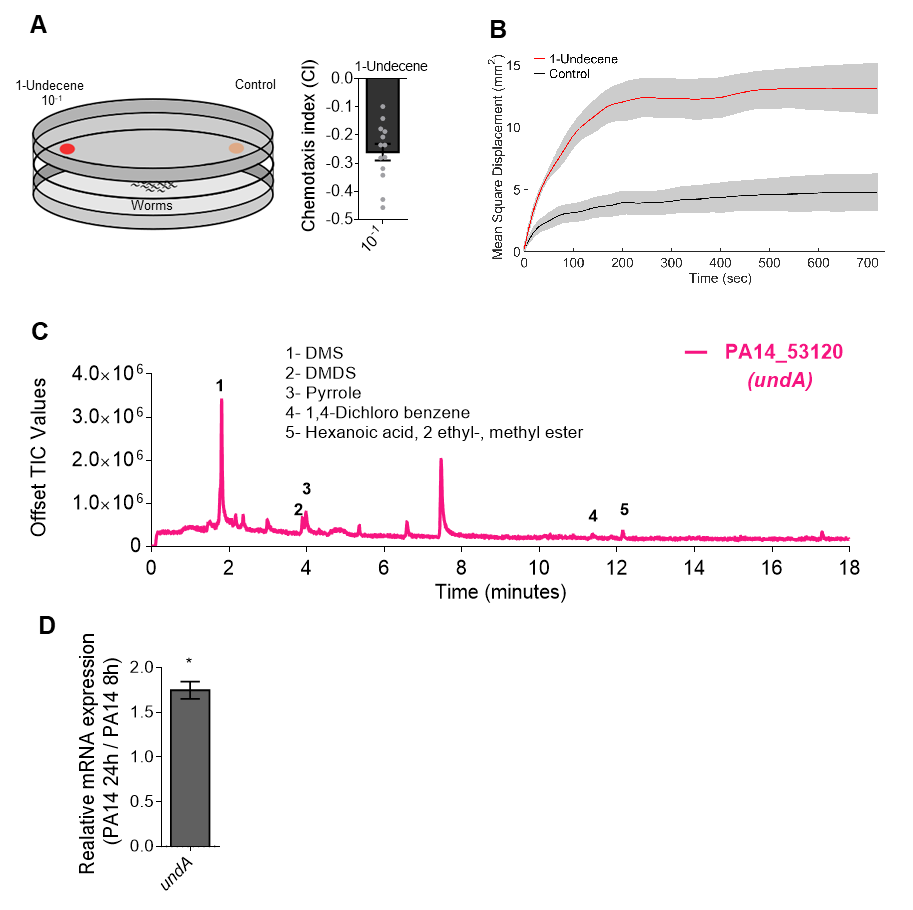


**Appendix Figure S2. 1-Undecene is sensed as a repellent and elicits defense responses in *C. elegans* (Related to Figures 2 and 3).**

1. Chemotaxis response of N2 worms for 1-undecene odor at 1:10 dilution. n ≥ 3 assays. Error bars indicate SEM.
2. Mean square displacement (MSD) of worms under exposure of 1-undecene and control condition. MSD is defined by〈δ*r*^2^(*t*)〉∝ *t^μ^*  where δ*r* is displacement of worms in time t and the slope of double logarithm MSD curve is defined by μ. The μ for control is 0.7144 and μ under 1-undecene exposure is 0.9216 (detail in methods).
3. GC-MS/MS profile of volatiles produced by 24 h old lawn of *undA* mutant of *P. aeruginosa*.
4. Real time PCR analysis of *undA* gene in old lawn of PA14 (24 h) over young lawn of PA14 (8h). n ≥ 3 assays. * P ≤ 0.05 as determined by two-tailed unpaired t-test. Error bars indicate SEM.

**Appendix Figure S3** **(related to Figure 3)**

**Fig. S3. 1-Undecene is sensed by AWB odor sensory neurons of *C. elegans*** **(Related to Figure 3).**

Average calcium responses of transgenic worms expressing GCaMP family of indicator in AWA, AWB AWC^on^ and AWC^off^ neurons recorded for 180 s under 1:100 dilution of 1-undecene.

**Appendix Figure S4** **(related to Figure 4)**


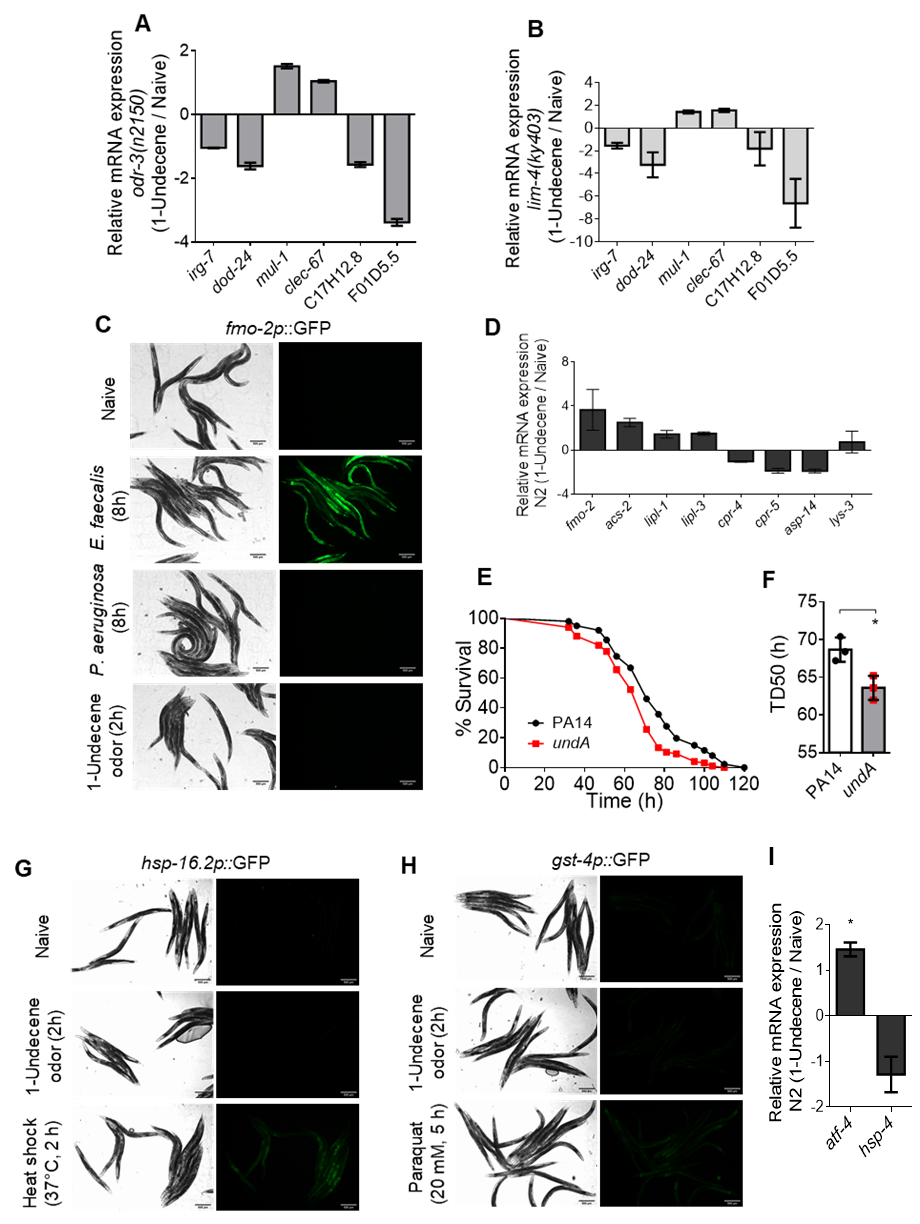


**Appendix Figure S4. 1-Undecene odor induces pathogen-specific immune response in *C. elegans.* (Related to figure 4)**

1. Real time PCR analysis of *P. aeruginosa* specific immune response genes in *odr-3(n2150)* worms exposed to 1-undecene odor upon naive *odr-3(n2150)* worms. n = 3. Error bars indicate SEM.
2. Real time PCR analysis of *P. aeruginosa* specific immune response genes in *lim-4(ky403)* worms exposed to 1-undecene and naive *lim-4(ky403)* worms. n = 3. Error bars indicate SEM.
3. *fmo-2p*::GFP induction in worms exposed to *E. coli* OP50 (naive), *E. faecalis, P. aeruginosa and E. coli* OP50 under 1-undecene odor exposure. Scale bar = 500 um.
4. Real time PCR analysis of *E. faecalis* specific immune response genes in N2 worms exposed to 1-undecene odor upon naive N2 worms. n = 3. Error bars indicate SEM.
5. Kaplan Meier survival curve of N2 worms on *P. aeruginosa* wild type (PA14) and *undA* mutant. Survival assay was performed at 20°C.
6. Time required for 50% of worms to die (TD_50_) on *P. aeruginosa* wild type (PA14) and *undA* mutant*.* Each data point indicate replicates with ~100 worms. n = 3 assays. * P ≤ 0.05 as determined by two-tailed unpaired t-test. Error bars indicate SEM.
7. *hsp-16.2*p::GFP induction in worms exposed to *E. coli* OP50 (naive), 1-undecene odor and heat shock. Scale bar = 500 um.
8. *gst-4*p::GFP induction in worms exposed to *E. coli* OP50 (naive), 1-undecene odor and 20mM paraquat. Scale bar = 500 um.
9. Real time PCR analysis of *atf-4* and *hsp-4* genes in N2 worms exposed to 1-undecene odor upon naive N2 worms. n = 3. Error bars indicate SEM.

**Appendix Table S1:** *C. elegans* strains used in this study

| **Strain Name** | **Genotype** | **Name and Figures** |
| --- | --- | --- |
| CX2205 | *odr-3(n2150)* | Figures 1A-1F, 3A, 4D, and S4A |
| MT4810 | *odr-3(n2046)* | Figures 1A, 1C-1E, 3B and 4D |
| CX4 | *odr-7(ky4)* | Figure 3B |
| AWA(-) | agEx [odr-10p::TU#813 + odr- 10p::TU#814 + unc-122p::GFP]. AWA ablated | Figure 3B |
| ASH(-) | agEx [sra-6p::TU#813 + del-2p::TU#814 + unc-122p::GFP]. ASH ablated | Figure 3B |
| CX3937 | *lim-4(ky403)* | Figures 3B, 4E and S4B |
| JY359 | *lim-4(yz12)* | Figures 3B and 4E |
| PY7502 | oyIs85 [ceh-36p::TU#813 + ceh 36p::TU#814 + srtx-1p::GFP + unc-122p::DsRed]. AWC ablated | Figure 3B |
| PY6554 | oyEx [gpa-4::GCaMP2.2b, unc-122::dsRed] | AWA; Figure S3A |
| PY7336 | \| oyEx [str-1::GCaMP3, unc-122::dsRed] \| \| --- \| | AWB; Figures 3C-3F and S3A |
| CX10536 | \| kyEx2595 [str-2::GCaMP2.2b, unc-122::gfp] \| \| --- \| | AWC^ON^; Figure S3A |
| PY6253 | \| pha-1(e2123) III; syEx1238 [srsx-3::GCaMP3, pha-1::pha-1] \| \| --- \| | AWC^OFF^; Figure S3A |
| ERT61 | *zip-2(tm4248)* | Deletion allele, Figure 4B  (Emily Troemel’s lab) |
| AU133 | agIs17 [myo-2p::mCherry + irg-1p::GFP] IV | Figure 4C |
| *fmo*-2GFP | Pfmo-2::GFP | Figure S4C |
| CL2070 | *hsp-16.2p::GFP* | Figure S4G |
| CL2166 | *gst-4p::GFP* | Figure S4H |
