# Supplementary material for: 1‐Undecene from Pseudomonas aeruginosa is an olfactory signal for flight‐or‐fight response in Caenorhabditis elegans
Source: EMBO J. 2021 Jun 4;40(13):e106938. doi: 10.15252/embj.2020106938 (PMC8246062; doi:10.15252/embj.2020106938)
Supplement: Supplementary file 2 — Movie EV1 [file EMBJ-40-e106938-s003.zip › Movie EV1.docx]

**Movie EV1**: S1a- Avoidance response of wild type worms on *P. aeruginosa* lawn for a period of 12h; speed of the video- 600 X. S1b- Avoidance response of *odr-3(n2150)* mutant worms on *P. aeruginosa* lawn for a period of 12h; speed of video- 600 X.
