# Supplementary material for: 1‐Undecene from Pseudomonas aeruginosa is an olfactory signal for flight‐or‐fight response in Caenorhabditis elegans
Source: EMBO J. 2021 Jun 4;40(13):e106938. doi: 10.15252/embj.2020106938 (PMC8246062; doi:10.15252/embj.2020106938)
Supplement: Supplementary file 3 — Movie EV2 [file EMBJ-40-e106938-s005.zip › Movie EV2.docx]

**Movie EV2**: S2a- Chemotaxis response of wild type worms towards 1-undecene (10-1 ); speed of the video- 200 X. S2b- Chemotaxis response of *odr-3(n2150)* mutant worms towards 1-undecene (10^-1^); speed of the video- 200 X.
