# Supplementary material for: 1‐Undecene from Pseudomonas aeruginosa is an olfactory signal for flight‐or‐fight response in Caenorhabditis elegans
Source: EMBO J. 2021 Jun 4;40(13):e106938. doi: 10.15252/embj.2020106938 (PMC8246062; doi:10.15252/embj.2020106938)
Supplement: Supplementary file 4 — Movie EV3 [file EMBJ-40-e106938-s001.zip › Movie EV3.docx]

**Movie EV3**: Wild type worms showing reversal and omega turn when exposed to 1- undecene; speed of the video- 2X.
